# Victimisation of individuals with serious mental illness living in sheltered housing: differential impact of risk factors related to clinical and demographic characteristics

**DOI:** 10.1192/bjo.2021.57

**Published:** 2021-05-06

**Authors:** Milan Zarchev, Cornelis L. Mulder, Jens Henrichs, Diana P. K. Roeg, Stefan Bogaerts, Jaap van Weeghel, Astrid M. Kamperman

**Affiliations:** Department of Psychiatry, Erasmus University Medical Center, The Netherlands; Department of Psychiatry, Erasmus University Medical Center, The Netherlands; Department of Midwifery Science, Amsterdam University Medical Center, The Netherlands; Research Division, Kwintes Supported Housing The Netherlands; and Tranzo, Tilburg School of Social and Behavioral Sciences, Tilburg University, The Netherlands; Department of Developmental Psychology, Tilburg School of Social and Behavioral Sciences, Tilburg University, The Netherlands; Tranzo, Tilburg School of Social and Behavioral Sciences, Tilburg University, The Netherlands; Department of Psychiatry, Erasmus University Medical Center, The Netherlands

**Keywords:** Victimisation, serious mental illness, sheltered housing, supported housing, living conditions

## Abstract

**Background:**

Sheltered housing is associated with quality-of-life improvements for individuals with serious mental illness (SMI). However, there are equivocal findings around safety outcomes related to this type of living condition.

**Aims:**

We aimed to investigate raw differences in prevalence and incidence of crime victimisation in sheltered housing compared with living alone or with family; and to identify groups at high risk for victimisation, using demographic and clinical factors. We do so by reporting estimated victimisation incidents for each risk group.

**Method:**

A large, community-based, cross-sectional survey of 956 people with SMI completed the Dutch Crime and Victimisation Survey. Data was collected on victimisation prevalence and number of incidents in the past year.

**Results:**

Victimisation prevalence was highest among residents in sheltered housing (50.8%) compared with persons living alone (43%) or with family (37.8%). We found that sheltered housing was associated with increased raw victimisation incidence (incidence rate ratio: 2.80, 95% CI 2.36–3.34 compared with living with family; 1.87, 95% CI 1.59–2.20 compared with living alone). Incidence was especially high for some high-risk groups, including men, people with comorbid post-traumatic stress disorder and those with high levels of education. However, women reported less victimisation in sheltered housing than living alone or with family, if they also reported drug or alcohol use.

**Conclusions:**

The high prevalence and incidence of victimisation among residents in sheltered housing highlights the need for more awareness and surveillance of victimisation in this population group, to better facilitate a recovery-enabling environment for residents with SMI.

Following the deinstitutionalisation of psychiatric services, individuals with serious mental illness (SMI) increasingly live within the community. Broadly, two housing strategies have been developed, in the hopes of facilitating recovery by integrating SMI individuals in the community.^1^ The options consist of living independently by making use of out-patient support services or living in a sheltered housing arrangement, sharing some spaces with others and having staff that are available during working hours or overnight.^2^ Although different terms are used in the literature (e.g. sheltered, supported, recovery housing), we discuss sheltered housing as defined by persons with SMI who live in shared community or halfway housing. The living conditions and therapeutic environment of sheltered housing in The Netherlands, the country of this study, are very similar to those in other Western countries.^3,4^ This housing arrangement exists to provide people with SMI with an opportunity to reside within the general community and retain the benefits of a semi-monitored environment.^5^ Sheltered housing has received much attention from studies investigating its merits, but less from quantitative investigations.^6^

## Equivocal outcomes linked to sheltered housing

Research has found produced equivocal results on the quality and experience of sheltered housing. There appears to be a positive trend in quality-of-life outcomes, including better social functioning and living conditions, compared with alternative high-support accommodations, including the hospital.^6–8^ More-focused qualitative research suggests that these trends are attributable to residents feeling safe and distancing themselves from past stress and trauma.^9,10^ At the same time, recommendations have been made for widespread implementation of sheltered housing in European and international psychiatric rehabilitation programmes.^11,12^

However, literature has also reported on high crime victimisation prevalence (25%) among English people with SMI who reside in sheltered housing.^13^ Longitudinal follow-up on the safety of sheltered housing found that the enthusiasm of newfound security faded throughout the first year of residence, as over time, substance misuse relapses and conflicts with neighbours reintroduced stress and mistrust among residents with SMI.^14^ This potentially reflects the general tendency for vulnerable populations to be housed in less-secure urban areas, satirically self-described in some contexts as ‘mental illness ghettos’ by tenants.^14,15^ Victimisation is likely to lead to worsened symptoms and adverse mental health, thus disrupting the recovery-focused goals upheld by the sheltered housing initiatives.^16^

## Gaps in the quantitative sheltered housing literature

These reports have stimulated commentary, inviting more quantitative research into the quality indicators and outcomes associated with sheltered housing.^17^ Although the literature so far describes in rich qualitative detail the experiences of individuals with SMI, very little research is available to quantify the threats associated, especially with regard to identifying demographic or clinical groups at increased risk of adversity. Gender is a prominent example of a factor often identified in forensic research as particularly relevant to victimisation and its complex implication on the consequences that follow.^18^ Unfortunately, little such information is available on whether residents of sheltered housing report different degrees of victimisation according to gender, or across other basic demographic factors commonly studied in victimisation literature (e.g. age, education; see de Vries et al^19^). As patients with SMI are at a much higher risk of becoming victims of crimes, there is potential for these risk factors to produce particularly salient differences in victimisation outcomes.^20^ Considering that inhabitants describe safety as the most important factor for their well-being at home, it is important to identify risk groups and establish the incidence and prevalence of crime victimisation for persons with SMI who are living in sheltered housing.^14,21^

## Aims

The current large-scale, cross-sectional study aims first to investigate differences in prevalence and incidence of victimisation in the past year in patients with SMI across three different types of living accommodation: living with family, living alone and living in sheltered housing. Furthermore, we also investigated which clinical and demographic variables are potential risk factors for victimisation, particularly emphasising potential gender differences.

## Method

### Design

The current study is embedded in the Victimization in Psychiatric Patients study, a cross-sectional epidemiological survey of a large, random community sample of 956 patients with SMI in The Netherlands.^20^ This study was approved by the Medical Ethics Committee of the Erasmus Medical Center, Rotterdam (approval number MEC-2010-232). Written informed consent was obtained from all participants.

### Participants

Eligible for the study were women and men, aged between 18 and 65 years, who were out-patients of six mental healthcare institutions in urban or rural areas of The Netherlands. They had to be diagnosed with a psychotic, bipolar or major depressive disorder. Excluded were patients with insufficient command of the Dutch language, who were incarcerated in prison or who were unable to answer study questions because they were experiencing acute symptoms.^20^ Enrolment occurred between December 2010 and April 2012.

### Procedures

As previously depicted by Kamperman et al,^20^ data on crime victimisation and clinical and demographic variables were obtained in a structured, computer-assisted, face-to-face interview. Respondents received a €20 cash incentive at the end of the interview. Interviews took 75 min on average (range 40–160 min), and were carried out at the respondents’ discretion in their home or at the mental healthcare institution. Interviewers were master's level social scientists with training in conducting the interviews with patients with SMI, as supervised by an experienced coordinator.

### Instruments

#### Main determinant and risk factors

Participants self-indicated whether they lived independently or in a sheltered housing arrangement, with the latter category including any recovery-focused halfway or community housing for out-patients of SMI services. Those who lived independently were subdivided into living alone or living with family, which included a partner, child or member of extended family. Those who indicated living in sheltered housing provided an address, which was cross-checked to validate a sheltered housing establishment in fact operates on that address. Such establishments can vary in the number of people sharing space and degree of monitoring by staff. No additional data was available on these specifics for those who indicated living in sheltered housing.

Clinical characteristics were operationalised with standardised instruments. Symptoms of post-traumatic stress disorder (PTSD) were assessed with the Self-Rating Inventory for Posttraumatic Stress Disorder.^22^ Perpetration of physical violence over the past year was assessed by the physical assault subscale (12 items) of the Conflict Tactics Scale short form.^23^ The Dimensions of Anger Reactions Scale^24^ was used to assess trait anger. Substance misuse was assessed with the Dutch version of the 12-month drug and alcohol use questionnaire of the European Monitoring Centre for Drugs and Drugs Addiction.^25^ For this study, we operationalised alcohol misuse as drinking more than six consumptions per day at least one time over the past 6 months. Drug misuse was operationalised as using one or more types of drugs, or using medication without a doctor's prescription. All instruments exhibited good reliability (Cronbach's *α*>0.80). See the Supplementary Material available at https://doi.org/10.1192/bjo.2021.57 for detailed information on dichotomisation, reliability and construct validity for each clinical instrument.

Sociodemographic characteristics were collected on gender, age, ethnicity, marital and employment status and educational level. Following the definition of the Dutch government,^26^ ethnicity was classified on the basis of country of birth and parents’ country of birth.

#### Outcome

Prevalence of crime victimisation and the number of incidents in the past year were assessed with the crime victimisation scale of the Dutch Crime and Victimisation Survey (*Integrale Veiligheidsmonitor*; IVM).^27^ The IVM crime victimisation scale strongly resembles the International Crime Victimization Survey.^28^ The IVM crime victimisation scale consists of 14 screening questions on various types of property crime, personal crime and vandalism. These include burglary, attempted burglary, bicycle theft, pick-pocketing, robbery, theft (other), vandalism (other), sexual harassment or assault, threatened with violence, physical assault and crime (other). For each reported incident in the preceding year, detailed information on the time and number of incidents, setting and perpetrator is assessed. To minimalize the effect of telescoping, the respondents are asked to recall incidents over the past 5 years, before recalling incidents over the past year. There are no traditional reliability and validity scores of the IVM crime victimisation scale.^27^

### Statistical analysis

The main determinant of the current analysis was the living condition factor and its predictive value on victimisation. As such, we report on univariable, multivariable and interaction coefficient terms related to the living conditions predictor. A multivariable Poisson regression was conducted to investigate the role of living conditions on estimating victimisation. We utilised a logit link to model the count outcome, using the ‘glm’ package developed by the R core team (R, version 3.6.2 for Windows, R Foundation for Statistical Computing, Vienna, Austria; see https://www.R-project.org/). In reporting on the multivariable Poisson regression consisting of multiple interaction terms, we present effect plots of estimated number of victimisation incidences to facilitate intuitive interpretation of risk groups identified in the model, instead of focusing exclusively on coefficient values, which are difficult to interpret, if at all meaningful.^29^ The reported results were stratified by gender, to meaningfully inspect differences between genders. All reporting on significant differences in estimated incidents are based on 95% confidence intervals.

A model-building approach was taken in understanding the effect of living conditions on victimisation incidence. We used a logistic regression for unadjusted prevalence. Next, we estimated a univariable Poisson regression model to investigate the unadjusted association between living conditions and victimisation incidence. This univariable model contained dummy-coded living conditions variables corresponding to a sheltered housing versus family accommodation comparison and living alone versus family accommodation comparison. In a second step, a full multivariable logistic model was fitted, to include gender, education, comorbid drug use in the past year, comorbid alcohol misuse in the past 6 months, comorbid PTSD, perpetration of assault in the past year and dispositional anger. These variables were chosen on a theoretical basis, as the literature has previously identified them as relevant to victimisation.^19^ They were also modelled as interactions with the living conditions dummy variables, to investigate their effect specifically within each living condition. Additionally, age, marital status and employment were included only as covariates. In the last step of variable selection, we applied a backward-model selection based on the Akaike information criterion (AIC), to find a more parsimonious set of variables explaining the data. We chose the AIC over other information criteria, like the Bayesian information criterion (which produced identical results), as it is relatively liberal in allowing model complexity.^30^

In total, 5% of data were missing; 52 cases had missing data. Five variables accounted for missing data: alcohol misuse (*n* = 10; 1.0% of cases), comorbid PTSD (*n* = 11; 1.2% of cases), perpetration of violence (*n* = 13; 1.4% of cases), housing (*n* = 29; 3.0% of cases) and dispositional anger (*n* = 10, 1.0% of cases). Missing data patterns were explored to assess whether the missing-at-random assumption was met for the victimisation outcome, before dropping the missing data from further analysis. Descriptive plots of missing data patterns are presented in Supplementary Figure 1. Multicollinearity between predictor variables was assessed with variance inflation factors (>2.0), as implemented in the ‘car’ package.^31^ Six cases were identified as outliers, reporting extremely high numbers of victimisation incidents (>100), and were additionally removed from analysis.

## Results

### Sample

The full sample consisted of 956 patients with SMI: 608 men (64%) and 348 women (36%). Mean age was 44.7 years (s.d. 10.4). The respondents’ demographic and clinical characteristics were consistent with nationwide figures for patients with SMI in The Netherlands.^32^ Further demographic characteristics of the sample used for the main analysis are presented in Table 1.

**Table 1:** Demographic characteristics of the sample included in the analysis

	Overall, *N* = 898	Sheltered housing, *n* = 191	Living with family, *n* = 233	Living alone, *n* = 474
Gender				
Men	565 (63%)	146 (76%)	112 (48%)	307 (65%)
Women	333 (37%)	45 (24%)	121 (52%)	167 (35%)
Age, years				
18–30	95 (11%)	27 (14%)	36 (15%)	32 (6%)
31–40	217 (24%)	45 (24%)	60 (26%)	112 (24%)
41–50	282 (31%)	66 (35%)	65 (28%)	151 (32%)
51–65	304 (34%)	53 (28%)	72 (31%)	179 (38%)
Education status				
Low	200 (22%)	56 (29%)	47 (20%)	97 (20%)
Mid-low	308 (34%)	74 (39%)	74 (32%)	160 (34%)
Mid-high	251 (28%)	50 (26%)	75 (32%)	126 (27%)
High	139 (15%)	11 (5.8%)	37 (16%)	91 (19%)
Marital status				
Single	518 (58%)	135 (71%)	58 (25%)	325 (69%)
Relationship	221 (25%)	26 (14%)	149 (64%)	46 (10%)
Divorced/widow	159 (18%)	30 (16%)	26 (11%)	103 (21%)
Employment				
Unemployed	768 (86%)	180 (94%)	185 (79%)	403 (85%)
Employed	130 (14%)	11 (6%)	48 (21%)	71 (15%)
Ethnicity				
Non-Dutch	342 (38%)	79 (41%)	103 (44%)	160 (34%)
Dutch	556 (62%)	112 (59%)	130 (56%)	314 (66%)

### Prevalence and incidence, univariable and multivariable effects

In the first step of the analysis, investigating the unadjusted prevalence and incidence revealed that persons using sheltered housing services have the highest victimisation prevalence (50.8%, 95% CI 43.7–57.9%), followed by persons living alone (43.0%, 95% CI 38.6–47.5%) and persons living with family (37.8%, 95% CI 31.5–44.0%). Compared with persons living with family, persons living in sheltered housing or living alone reported significantly more victimisation incidents in the past year, at a higher incidence rate of 2.80 (95% CI 2.36–3.34) and 1.87 (95% CI 1.59–2.20) times, respectively (both *P* < 0.001), as shown in Table 2.

**Table 2:** Prevalence, incidence and univariable effects on incidence of living conditions on victimisation in the past year, in the current sample

	*n/N*	Prevalence	Incidence^a^	IRR [s.e.]^b^
Total		(1 year)	(1 year)	
Living with family	88/233	37.8%	0.79	—
Sheltered housing	97/191	50.8%	2.21	2.80 [0.09]^c^
Living alone	204/474	43.0%	1.87	1.87 [0.08]^c^
Men				
Living with family	36/112	31.6%	0.60	—
Sheltered housing	73/146	50.0%	2.23	3.73 [0.13]^c^
Living alone	133/307	43.3%	1.26	2.10 [0.13]^c^
Women				
Living with family	52/121	43.0%	0.97	—
Sheltered housing	24/45	53.3%	2.16	2.23 [0.14]^c^
Living alone	71/167	42.5%	1.87	1.94 [0.11]^c^

IRR, incidence rate ratio.

a. Calculated per person by dividing total number of incidents within group by group size

b. Reference group is living with family, stratified within each gender.

c. Significant at *α* = 0.001.

Following the strong differences in prevalence and incidence between the living condition categories, we used a multivariable Poisson regression to identify high-risk groups. We included gender, education, comorbid drug use in the past year, comorbid alcohol misuse in the past 6 months, comorbid PTSD, perpetration of physical assault in the past year and dispositional anger as both predictors and interaction terms with the living condition term. Additionally, age, marital status and employment status were included as confounder variables. The backward-model selection approach revealed that removing the employment variable and dispositional anger interaction term produced the model with the relatively lowest AIC. This model was assumed as the best fit to the data, and results hereafter are reported from it.

When comparing shelter with family accommodation, we observed a significant interaction effect between shelter and family accommodation and gender (*P* < 0.001), education (*P* < 0.001), comorbid alcohol misuse in the past 6 months (*P* < 0.001), assault perpetration in the past year (*P* = 0.026) and comorbid PTSD (*P* = 0.015). Comparing living alone with living with family produced similar significant interactions, but the living conditions and comorbid drug misuse interaction was also significant (*P* < 0.001). The implications of these interactions is probed next, using estimated incidents for each category. A full coefficient table and plot for the final model are presented in Supplementary Table 1 and Supplementary Figure 2.

**Fig. 1:**
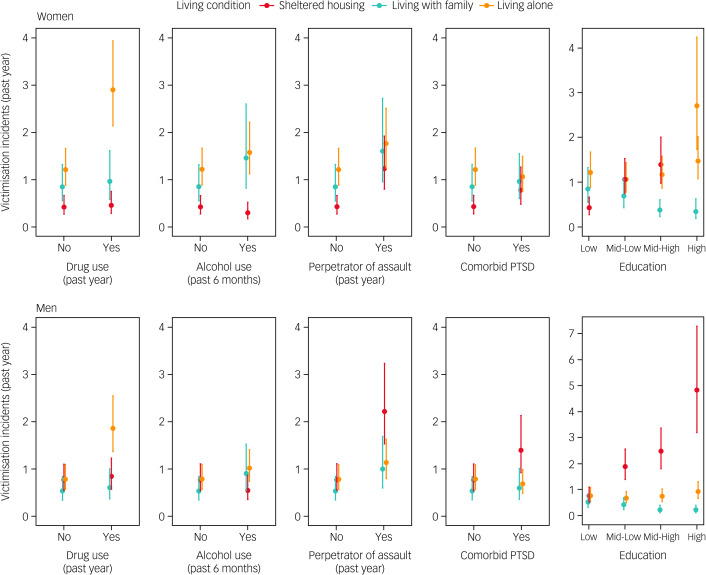
Estimated number of victimisation incidents in the past year, from multivariable Poisson regression for each person within a clinical or demographic risk group. The reference consists of individuals with no drug or alcohol misuse, no comorbid PTSD, no perpetration of assault and low education background. Each panel shows predicted victimisation after changing a single predictor (e.g. adding drug misuse to reference in the top left panel). PTSD, post-traumatic stress disorder.

Next, to examine the interactions with the living condition determinant, we report on the estimated incident counts obtained from the multivariable incidence model, visualised in Figure 1. It was estimated that women living alone reported the most incidents of victimisation in the past year (estimated victimisation incidents of 1.21, 95% CI 0.88–1.66), significantly more than those in sheltered housing (0.43, 95% CI 0.27–0.66). Victimisation incidents of those living with family were between the other two groups (0.853, 95% CI 0.55–1.32). Women with a comorbidity of drug misuse were estimated to be considerably more victimised when living alone (2.90, 95% CI 2.14–3.94). When reporting alcohol misuse, women became more likely to be victimised when living with family (1.46, 95% CI 0.82–2.61) than when living in sheltered accommodation (0.30, 95% CI 0.17–0.52). Next, women who self-reported perpetrating assault had more estimated victimisation incidents in sheltered housing than non-perpetrators (1.24, 95% CI 0.80–1.92), but having slightly lower victimisation estimates than those perpetrators living with family (1.61, 95% CI 0.96–2.72) or alone (1.77, 95% CI 1.24–2.51). Comorbid PTSD exhibited similar equivalence across all living conditions, thus all victimisation estimates were around 1 incident in the past year (approximate 95% CIs of 0.5–1.5). Finally, clear trends in victimisation were found across education categories, such that more-educated participants were at more risk of becoming victims in sheltered accommodation, and were at less risk when living with family. For instance, highly educated women in sheltered housing were estimated to report 2.70 incidents (95% CI 1.73–4.22), whereas the estimate when living with family was lower, at 0.34 (95% CI 0.19–0.63).

When examining the estimates for male patients, we found that in the reference category, there were no big differences between living with family (0.52, 95% CI 0.33–0.83), living alone (0.77, 95% CI 0.55–1.08) and living in a sheltered home (0.76, 95% CI 0.53–1.10). As with women, men were especially at risk of victimisation in the past year if they reported drug use in the past 6 months and lived alone (1.85, 95% CI 1.36–2.53). However, victimisation in sheltered living became much likelier for men if they had comorbid PTSD (1.39, 95% CI 0.91–2.12), were perpetrators of assault (2.21, 95% CI 1.51–3.22) and especially if they were highly educated (4.82, 95% CI 3.20–7.26). Again, there was a trend of less victimisation for educated men living with their family. Highly educated men were estimated to have 0.21 (95% CI 0.11–0.41) incidents in the past year when living with family, the lowest estimate to emerge from the analysis.

## Discussion

The current study investigated the association between living in sheltered housing and becoming a recent victim of crime in a large-scale community sample of patients with SMI. Several distinct patterns of results were revealed by the analysis. First, the highest overall victimisation prevalence was reported by those living in sheltered housing, where half of the patients reported becoming a crime victim at least once in the past year, as compared with 38% if living with family or 43% if living alone. Regarding incidence rates, a striking trend was that the more educated patients were, the more likely they were to be victimised in the sheltered housing category, and especially so for men. Victimisation among sheltered housing residents was also particularly high for men with PTSD or if they were a perpetrator of assault themselves. Women were less victimised when living in sheltered housing compared with men, often reporting less incidents when living there than when living alone or with family. Finally, both men and women living alone had a higher risk of being victimised if reporting alcohol or drug problems in the past year.

Previous research has reported mainly on positive outcomes linked to patients with SMI living in sheltered housing.^8^ A direct comparison of the current results is possible with a previous report on victimisation of patients with SMI in The Netherlands, which reported – in contrast to our study – that those living in sheltered housing were comparatively less victimised than alternative forms of out-patient accommodations, and indeed less than the prevalences reported here (27.6%).^33^ There are two potential reasons for this discrepancy with the current sample. First, the patients in that sample reported considerable substance use, almost half in the sheltered housing group (47% compared with 34% in our sample). This highlights the present finding that sheltered housing appears to be associated with less victimisation for those with drug and alcohol problems when compared with more deinstitutionalised settings, like living alone or with family. Additionally, however, the sample size of that study was much lower than that of the current study. Considering the current study only had a relatively small proportion of highly educated individuals, an even smaller study would not be able to pick up on the relatively rare, yet large effect of being highly educated in sheltered housing.^34,35^ Two hypotheses on the effect have been offered: educated people make more attractive targets because of perceived higher status,^36^ or alternatively, they are more ready to define an event as a crime.^37^ Because of small numbers in the current study and the lack of previous verification of the abovementioned hypothesis, this relationship needs further study.

Within SMI samples, much forensic research has been dedicated to investigating the association between the vulnerability of becoming a victim of crime and clinical factors like PTSD.^38^ No study so far had examined the implications for those living in sheltered housing. The current study links that line of research with the current research question by pinpointing male patients with SMI with comorbid PTSD as a particularly vulnerable subgroup. This finding is particularly troubling, as established theoretical models point to victimisation as an important mediator in how PTSD positively relates to worsened psychiatric symptoms.^16^ Thus, sensitivity to monitoring PTSD in sheltered housing is vital, as these individuals, and specifically men, are at a high risk of becoming victims of crime.

Finally, demographic factors were also found to be associated with how often patients were victimised when living in sheltered housing. A striking result of the current study was that women living in sheltered housing tended to be less vulnerable to crime victimisation in the past year compared with men. In general, women with SMI are more likely to become victims of family violence compared with men, who are more likely to be victims of crime perpetrated by non-familial offenders.^39^ The unfortunate reality of women being abused more by their own family is one explanation of the gender differences in the current study. The relatively depressed economic conditions around sheltered housing, a well-known risk factor for male victimisation, might further explain gender differences.^39^ These differences were especially evident for women reporting substance misuse, where victimisation prevalence was considerably lower in sheltered housing compared with living alone or with family. To a lesser extent, this was also true for men who reported substance misuse. Substance users tend to live in neighbourhoods with poor economic conditions, chronic disease and social disorganisation,^40^ which predisposes them to crime and trauma and worsens their mental health.^41^ This study provides clear evidence that sheltered housing is associated with less crime victimisation for individuals with SMI who have drug problems. Of note is that our model was adjusted for perpetration, therefore these victims were not also self-reported perpetrators. Future studies could thus establish sheltered housing as a potentially beneficial intervention for providing safety compared with the alternative, especially for women with drug or alcohol problems.

The foremost strength of the current study was its sample size: a representative random sample of many individuals was obtained, so that we could include rare characteristics of patients with SMI in our analysis (e.g. high education). Despite the many interaction terms included in our model, we could still obtain precise estimates for differences between each group. Robust as our results are, an important limitation is that they are confined to the sheltered housing system of The Netherlands, which can be heterogeneous itself in terms of monitoring and support. It is for future research to determine whether the findings are applicable in culturally and administratively different parts of the world, as well as within specific subcategories of sheltered housing. Additionally, the conclusions from a cross-sectional sample such as this one are correlational in nature, and thus prone to confounding. We do not make claims about the causal structure of the effects, nor can we establish in the current sample whether victimisation took place inside or outside of the housing premises. Nevertheless, this is the largest and most detailed study into the topic to date, allowing for robust prediction, if not causal understanding, of victimisation. There is potential for many further research questions to be formulated based on the current findings.

In conclusion, the current study highlights the need for higher awareness and better surveillance of victimisation among residents of sheltered housing, to better facilitate a recovery-enabling environment for patients with SMI. Individuals with higher education, and especially men with a PTSD diagnosis, are particularly vulnerable to becoming victims when living in sheltered housing. At the same time, individuals with comorbid substance misuse report less crime victimisation when living in sheltered housing, particularly so for women who have used drugs in the past year. Further intervention research is needed in this area, to inform on the specifics of how a monitoring and prevention intervention might work in practice. Although the current results provide insight into which people might need to receive increased attention, our study does not address differences across sheltered housing establishments. One area of research could focus on neighbourhood-level characteristics (e.g. economic deprivation level) and composition (e.g. exclusively female or targeting substance users) of sheltered housing establishments, and how they contribute to victimisation of patients with SMI. In light of the findings we have available, we conclude that the reality of sheltered housing and its implications for the crime victimisation of its inhabitants are more complex than can be captured by a purely positive or negative blanket statement. It can indeed be expected to provide a secure space suited for the recovery process of a given individual with SMI, but an important caveat to note is that some individuals inhabiting such accommodations are at a concerning risk of victimisation.

## Data Availability

Data are stored at the institutional database of the Erasmus Medical Centre in Rotterdam, The Netherlands. The data-sets on which the analyses are based are available on request to the Local Ethics Committee of the Erasmus Medical Centre in Rotterdam (https://doi.org/10.17026/dans-xht-ry34). The code used to analyse the current data-set is available in the Open Science Framework repository (https://osf.io/uw6hz/, DOI: 10.17605/OSF.IO/UW6HZ).

## Supplementary material

For supplementary material accompanying this paper visit http://dx.doi.org/10.1192/bjo.2021.57. click here to view supplementary material
