# Supplementary material for: Victimisation of individuals with serious mental illness living in sheltered housing: differential impact of risk factors related to clinical and demographic characteristics
Source: BJPsych Open. 2021 May 6;7(3):e97. doi: 10.1192/bjo.2021.57 (PMC8142546; doi:10.1192/bjo.2021.57)
Supplement: Supplementary file 1 [file S2056472421000570sup001.docx]

Victimisation of individuals with serious mental illness living in sheltered housing.

# *Supplementary materials*

**Clinical instruments used for risk factors**

Substance abuse is assessed using the Dutch version of the 12-month drug and alcohol use questionnaire of the European Monitoring Centre for drugs and Drugs Addiction (EMCDDA) (Van Rooij, Schoenmakers et al. 2011). For this study we operationalized alcohol abuse as drinking more than 6 consumptions per day at least one time over the past 6 months. Drugs abuse was operationalized as using one or more types of drugs, or using medication without a doctor’s prescription.

Symptoms of PTSD are assessed using the Self-Rating Inventory for Posttraumatic stress Disorder (SRIPD) (Hovens, Bramsen et al. 2002). The questionnaire consists of 22 items, reflecting the 17 PTSD symptoms according to DSM-IV. The items are scored on a 4 point Likert scale, ranging from ‘no problem’ to ‘very severe problem’. A score above 52 points can be interpreted as the presence of PTSD. Sensitivity is 86% and specificity is 71%. Reliability for the current sample is good (Cronbach α ranges from 0.90 to 0.94); construct validity is satisfactory (Brewin, 2005).

Perpetration of physical violence over the past 12-months is assessed by the physical assault subscale (12 items) of the Conflict Tactics Scale short form (CTV2) (Straus, Hamby et al. 1996). The items ask about mild and severe experiences of violent perpetration and are answered on a 6-point scale with response options ranging from ‘once a year’ to ‘more than 20 times a year’. The score was dichotomised in ‘no experiences’, and ‘one or more experiences’.

The Dimensions of Anger Reactions scale (DAR) (Hawthorne, Mouthaan et al. 2006) is used to assess trait anger. The DAR consists of seven items assessing anger, frequency, intensity, duration, antagonistic expression, and impairment of work performance, interpersonal relationships, and personal health. In the Dutch version, the items are rated on a 5-point scale ranging from 0 ‘not at all’ to 4’very much’ (Nederlof, Hovens et al. 2009). Higher scores reflect higher dispositional anger. Test-retest reliability in a Dutch population is good (r=0,84); construct validity is satisfactory (Nederlof, Hovens et al. 2009). The score is dichotomised in a low and a high dispositional anger category using the median score as cut-off (score =12).

*
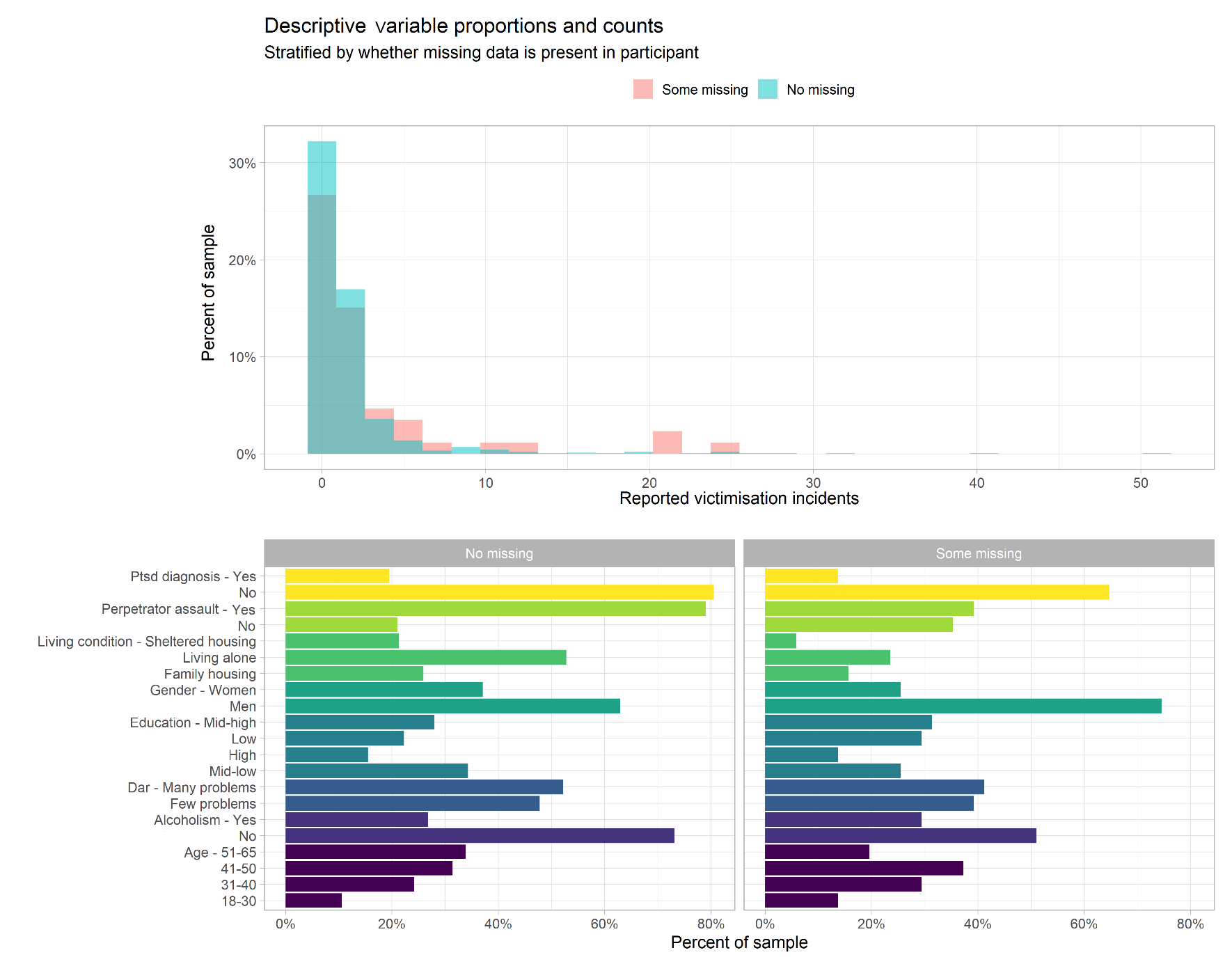
Supplementary Figure 1. Victimization incident counts (top) and proportions of participants in each covariate group (bottom), stratified by whether all data was present for participant or some was missing.*

| ***Supplementary Table 1. Final model effects and confounder coefficients from multivariable Poisson regression analysis*** | | | | | | | | | | |  |
| --- | --- | --- | --- | --- | --- | --- | --- | --- | --- | --- | --- |
|  | Estimate*^1^* | | | Std. Error | | 95 CI Lower | | Upper | | p-value |  |
| Sheltered Housing (vs. Family Household) | | | −0.69 | 0.27 | | −1.23 | | −0.15 | | .012 | |
| Single Person (vs. Family Household) | | | 0.35 | 0.22 | | −0.08 | | 0.80 | | .115 | |
| Drug use (past year) | | | 0.13 | 0.18 | | −0.24 | | 0.48 | | .473 | |
| Alcohol use (past 6 months) | | | 0.54 | 0.19 | | 0.16 | | 0.9 | | .004 | |
| Comorbid PTSD diagnosis | | | 0.12 | 0.16 | | −0.19 | | 0.43 | | .438 | |
| Perpetrator assault (past year) | | | 0.64 | 0.16 | | 0.32 | | 0.95 | | <.001 | |
| Dispositional anger | | | 0.43 | 0.06 | | 0.30 | | 0.55 | | <.001 | |
| Marital status (Single vs Divorced/Widowed) | | | −0.70 | 0.07 | | −0.84 | | −0.55 | | <.001 | |
| Marital status (Relationship vs Divorced/Widowed) | | | −0.52 | 0.10 | | −0.72 | | −0.33 | | <.001 | |
| Age 31-40 (vs. 18-31) | | | −0.07 | 0.11 | | −0.29 | | 0.15 | | .529 | |
| Age 41-50 (vs. 18-31) | | | 0.35 | 0.11 | | 0.15 | | 0.56 | | .001 | |
| Age 51-65 (vs. 18-31) | | | −0.11 | 0.11 | | −0.33 | | 0.12 | | .348 | |
| Gender (Women vs. Men) | | | 0.49 | 0.17 | | 0.16 | | 0.83 | | .004 | |
| Education (Mid-Low vs. Low) | | | −0.21 | 0.19 | | −0.59 | | 0.17 | | .275 | |
| Education (Mid-High vs. Low) | | | −0.81 | 0.22 | | −1.24 | | −0.39 | | <.001 | |
| Education (High vs. Low) | | | −0.91 | 0.29 | | −1.52 | | −0.35 | | .002 | |
| **Interaction effects with Shelter vs. Family Household** | | | | | | | | | | |  |
| Drug use (past year) | −0.04 | | | 0.22 | | −0.46 | | 0.38 | | .835 |  |
| Alcohol use (past 6 months) | −0.89 | | | 0.23 | | −1.33 | | −0.44 | | <.001 |  |
| Comorbid PTSD | 0.48 | | | 0.20 | | 0.09 | | 0.86 | | .015 |  |
| Perpetrator assault (past year) | 0.43 | | | 0.19 | | 0.05 | | 0.81 | | .026 |  |
| Gender (Women vs. Men) | -1.07 | | | 0.21 | | -1.49 | | -0.65 | | <.001 |  |
| Education (Mid-Low vs. Low) | 1.12 | | | 0.25 | | 0.63 | | 1.62 | | <.001 |  |
| Education (Mid-High vs. Low) | 2.00 | | | 0.27 | | 1.46 | | 2.54 | | <.001 |  |
| Education (High vs. Low) | 2.76 | | | 0.35 | | 2.08 | | 3.47 | | <.001 |  |
| **Interaction effects with Single vs. Family Household** | | | | | | | | | | |  |
| Drug use (past year) | 0.74 | | | 0.20 | | 0.36 | | 1.14 | | <.001 |  |
| Alcohol use (past 6 months) | −0.27 | | | 0.21 | | −0.67 | | 0.14 | | .184 |  |
| Comorbid PTSD | −0.26 | | | 0.18 | | −0.61 | | 0.11 | | .163 |  |
| Perpetrator assault (past year) | −0.26 | | | 0.18 | | −0.62 | | 0.10 | | .157 |  |
| Gender (Women vs. Men) | -0.04 | | | 0.19 | | −0.42 | | 0.33 | | .833 |  |
| Education (Mid-Low vs. Low) | 0.08 | | | 0.22 | | −0.35 | | 0.51 | | .721 |  |
| Education (Mid-High vs. Low) | 0.78 | | | 0.24 | | 0.3 | | 1.26 | | .001 |  |
| Education (High vs. Low) | 1.10 | | | 0.32 | | 0.5 | | 1.75 | | .001 |  |
| *^1^*Estimates are on the log-odds scale | | | | | | | | | | |  |

*
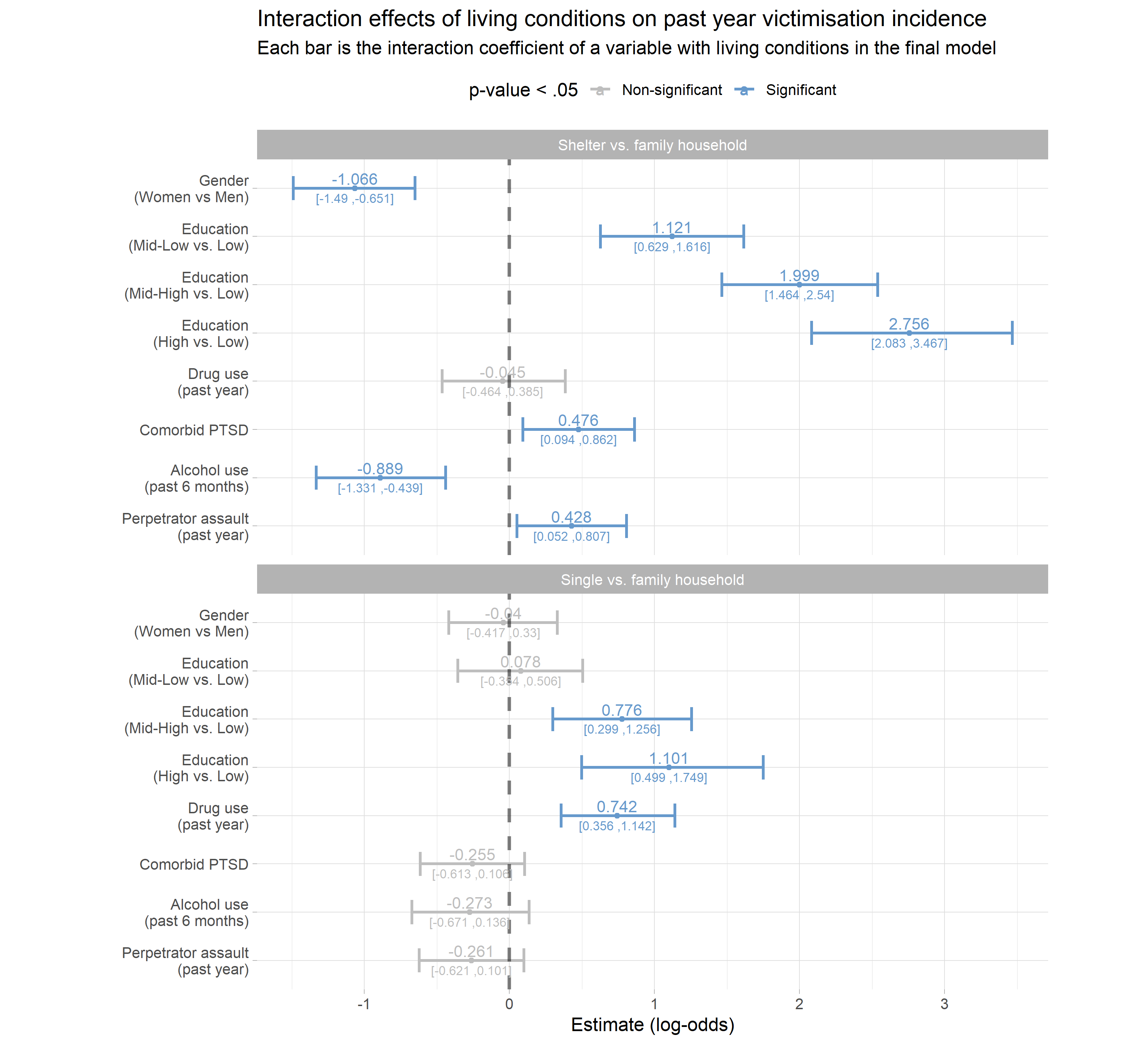
Supplementary Figure 2. Interaction effects of living conditions on past year victimization incidence. Each bar is the interaction coefficient of a variable (presented on the left) with the living conditions factor in the final model.*
